# Understanding the heterogeneity of childhood allergic sensitization and its relationship with asthma

**DOI:** 10.1097/ACI.0000000000000967

**Published:** 2024-02-07

**Authors:** Adnan Custovic, Darije Custovic, Sara Fontanella

**Affiliations:** National Heart and Lung Institute, Imperial College London, London, UK

**Keywords:** asthma, atopy, birth cohorts, childhood, heterogeneity, machine learning, prediction, rhinitis, sensitization, wheeze phenotypes

## Abstract

**Purpose of review:**

To review the current state of knowledge on the relationship between allergic sensitization and asthma; to lay out a roadmap for the development of IgE biomarkers that differentiate, in individual sensitized patients, whether their sensitization is important for current or future asthma symptoms, or has little or no relevance to the disease.

**Recent findings:**

The evidence on the relationship between sensitization and asthma suggests that some subtypes of allergic sensitization are not associated with asthma symptoms, whilst others are pathologic. Interaction patterns between IgE antibodies to individual allergenic molecules on component-resolved diagnostics (CRD) multiplex arrays might be hallmarks by which different sensitization subtypes relevant to asthma can be distinguished. These different subtypes of sensitization are associated amongst sensitized individuals at all ages, with different clinical presentations (no disease, asthma as a single disease, and allergic multimorbidity); amongst sensitized preschool children with and without lower airway symptoms, with different risk of subsequent asthma development; and amongst sensitized patients with asthma, with differing levels of asthma severity.

**Summary:**

The use of machine learning-based methodologies on complex CRD data can help us to design better diagnostic tools to help practising physicians differentiate between benign and clinically important sensitization.

## INTRODUCTION: HOW IS SENSITIZATION LINKED TO ATOPIC DISEASES?

There is broad scientific and clinical consensus that there exists an important association between allergic sensitization and atopic diseases (asthma, eczema, rhinitis and their comorbidities) [1]. However, the nature of this association is not fully understood. Three challenges in particular demonstrate this point well.

First, the question of how to accurately describe the association is unresolved; for example, ‘how much of asthma is attributable to atopy’ [2]?. In recent decades, numerous epidemiological studies have demonstrated close relationships between allergic sensitization and asthma and rhinitis (reviewed in [3–5]). However, the data about the strength of these associations vary considerably across studies [6–9], and there are no reliable and reproducible sensitization parameters on which to base accurate diagnosis and disease risk prediction [7,10,11].

Second, the aetiological link between sensitization and clinical presentation in some patients is uncertain. In clinical situations, the confirmation of sensitization using standard diagnostic tests (skin prick tests and/or measurement of sIgE) does not necessarily indicate that a patient's symptoms are *caused* by an IgE-mediated reaction [3,12^▪^]: in a proportion of patients with asthma and rhinitis, sensitization ascertained by standard tests may be a chance finding unrelated to the presence or severity of their lower or upper airway symptoms [13^▪^]. This has obvious ramifications for disease management [14].

Finally, imprecisions remain in our diagnostic methods for allergic sensitization. This is partly due to the common practice of defining sensitization as a binary variable (presence or absence) via often arbitrary cut-offs [15], resulting in excessive false positives. Quantifying allergic sensitization instead, by using IgE titre or size of skin test response, can increase specificity both in terms of diagnostic accuracy [16,17] and capacity to predict the persistence of symptoms [18]; however, a significant number of false-positive test results remain [3,4]. Consequently, although asthma is closely associated with allergic sensitization, most current guidelines do not recommend assessing allergic sensitization for asthma diagnosis or monitoring [19^▪^]; nonetheless, most physicians caring for asthmatic children consider monitoring allergy and assessment of sensitization status to be of very high or high priority when monitoring childhood asthma [20^▪▪^].

All these uncertainties about the role of sensitization in asthma and other atopic diseases may in part result from considering sensitization to be a single biological feature, agglomerating what may in fact be several subtypes of sensitization that differ in their associations with asthma, rhinitis and/or eczema [3]. In a finding consistent with this notion, a machine learning analysis with Bayesian inference which took into account the type of allergen, and the timing of onset and remission of IgE responses from infancy to school-age, suggested the existence of several distinct patterns of sensitization [21]. Further, one of these (described by authors as ‘multiple early atopic vulnerability’) was identified as a much stronger associate of asthma diagnosis than others, and amongst patients with asthma, as a marker of disease severity (including impaired lung function and high risk of severe exacerbations) [21,22]. Importantly, amongst sensitized children, lung function and airway hyperreactivity were poorer amongst those assigned to the multiple early class [21].

Assuming they exist, knowing which sensitization subtype an individual patient has, and how this will develop over their life course, may aid in predicting whether a sensitized individual will have asthma, what its severity will be, and whether allergic comorbidity will occur. However, at present, individuals can be assigned to different sensitization clusters only in retrospect, through the modelling of longitudinal data on allergy tests collected over years or even decades. What we need for diagnostic and prognostic purposes are biomarkers which differentiate, in individual sensitized patients – and preferably from a single clinical consultation – whether their sensitization is important for current and future symptoms, or whether it is a finding of little or no relevance to the disease. However, accurate prediction based on a biologically sound interpretation is further complicated by the heterogeneity of atopic diseases. 

**Box 1:**
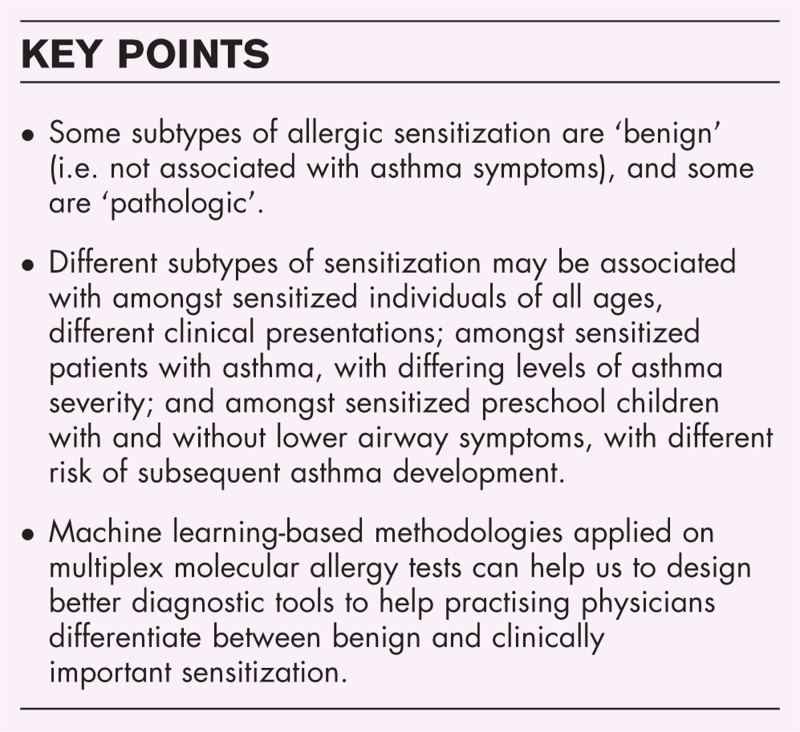
no caption available

## THE HETEROGENEITY OF ALLERGIC DISEASES

It has long been noted that atopic diseases (including asthma, eczema, rhinitis and multimorbidity) exhibit heterogeneity across patients, both in the time-course of the development/progression of symptoms (curricular heterogeneity) and their underpinning pathological mechanisms (aetiological heterogeneity) (reviewed in [23^▪▪^]). Since neither of these aspects is fully understood and clinical presentations in patients whose symptoms are caused by different mechanisms may nevertheless be similar, current diagnostic labels are imprecise: they are predominantly symptom and/or medication-based, rather than mapping cleanly onto underlying disease mechanisms [13^▪^]. It is consequently unsurprising that long-term prognoses remain uncertain.

To tackle these issues, substantial effort has been devoted over the last 15 years to understanding the heterogeneity of childhood atopic diseases using modern data approaches (reviewed in [23^▪▪^,^24^–^27^]). These data-driven analyses have revealed hidden/unobservable (latent) structures in the large longitudinal datasets from birth cohort studies; especially noteworthily, the methods have now described different clusters of wheeze/asthma [5,28^▪^,29^▪^] (reviewed recently in [23^▪▪^,^30^,^31^]), allergic multimorbidity [32^▪^], rhinitis [33], eczema [34^▪^,^35^] and lung function [36,37^▪^,^38^,39^▪^]. Important questions which still need to be answered are: (1) whether different clusters of asthma and atopic disease are mechanistically different diseases which are underpinned by different pathophysiological mechanisms [40]; and (2) in the context of this review, whether they differ in their associations with allergic sensitization.

In relation to the first point, if the genetic associates of these various clusters were found to be different, it would suggest that different mechanisms might be contributing to clinical presentation. The evidence tilts in this direction. One study suggested that the associations between 17q12–21 variants were similar in all childhood wheeze phenotypes, indicating a shared a genetic origin in relation to this locus [41]. In contrast, others have reported that variants at this locus are associated with increased risk of Persistent, but not Transient wheeze [42]. A recent large study which derived wheeze phenotypes from longitudinal, birth-to-adolescence data in more than 15 000 individuals, suggested that genetic associates are phenotype-unique [43^▪▪^]: by conducting a multivariate genome-wide association study (GWAS) of the wheezing phenotypes derived by data-driven methods, subsets of independent single nucleotide polymorphisms (SNPs) were found which were exclusively associated with the persistent wheeze, preschool remitting mid-childhood remitting or late-onset wheeze phenotypes [43^▪▪^]; little evidence was found of a shared genetic architecture between different phenotypes. The analysis also identified two GWAS-significant loci associated exclusively with persistent wheeze (but not any other wheeze phenotype): the aforementioned 17q12-21, *P* < 5.5 × 10^–9^, and a novel locus on chr 9q21.13 close to *annexin 1* (*ANXA1*), *P* < 6.7 × 10^–9^). Furthermore, functional studies in a mouse model demonstrated that both ANXA1 protein and mRNA expression were significantly increased in lung tissue following exposure to dust mite allergen, and experiments in ANXA1-/- deficient mice indicated that loss of ANXA1 resulted in increased airway hyperreactivity and T2 inflammation upon allergen challenge [43^▪▪^]. This series of experiments suggest that annexin 1 may be important in wheezing persistence via mechanisms associated with modulating responses to allergens. This raises the question as to whether different classes/clusters/phenotypes of asthma and other atopic diseases differ in their relationship with allergic sensitization.

## THE RELATIONSHIP OF ATOPIC DISEASE CLUSTERS TO SENSITIZATION

The relationship of different wheeze phenotypes to allergic sensitization (as it is usually measured) is complex. Despite distinct genetic architectures, longitudinal patterns of allergic sensitization across different wheeze cluster/phenotypes seem highly concordant, with trajectories of sensitization from infancy to adolescence being almost identical in persistent, intermittent and late-onset wheezing, while differing from those with no wheezing and early transient wheezing [28^▪^]. Meanwhile, *all* phenotypes of wheezing were associated with sensitization in early-school age, although the risk was higher in persistent and late-onset wheezing than transient wheezing [28^▪^]. Temporally, wheeze in general preceded sensitization in the persistent and intermittent clusters, while sensitization preceded wheeze in late-onset wheezing [28^▪^]. Similarly, overlapping sensitization trajectories were recently reported in relation to different lung function trajectories [39^▪^]. In complete contrast to these findings, similar unsupervised analysis in a South African birth cohort study has shown no association between persistent wheeze and sensitization [29^▪^].

Comparable with findings for different phenotypes of wheezing, very similar associations between allergic sensitization and different eczema clusters derived using data-driven methodologies were recently reported [34^▪^]. All eczema clusters were associated with allergic sensitization in early-school age (although the risk was highest for persistent eczema). Eczema preceded sensitization in the persisting clusters, and at age 1 year, less than 10% of children in the clusters with infantile eczema whose symptoms persisted to adolescence were sensitized; by age 16, approximately half were. In contrast, children with late-onset eczema tended to develop sensitization before the onset of eczema [34^▪^].

A recent study reported interesting data on the relationship between atopic multimorbidity and sensitization [32^▪^]. Children with multimorbidity (eczema, wheeze and rhinitis) were more likely to be sensitized than those with single diseases, and sensitization prevalence was consistently higher in the group with persistent multimorbidity. However, more than half of individuals with persistent ‘atopic’ multimorbidity were not sensitized at age 5 years, and nearly 30% were not sensitized in adolescence [32^▪^].

Finally, a hypothesis-generating analysis of data on lower airway inflammation and infection from bronchoalveolar lavage in preschool children with severe wheeze suggested the existence of four pathophysiological clusters, which had distinct allergic sensitization profiles and blood eosinophils, and also differed in BAL microbial profiles [44]. One of these clusters very strongly mapped to sensitization, but it comprised only a quarter of children with severe wheezing [44].

Taken together, this relative lack of clarity and consistency on the role of sensitization in asthma and atopic disease, both among individual patients and at the population level, is a further indication that the term ‘allergic sensitisation’ as used currently in clinical practice likely amalgamates several distinct subtypes of sensitization that differ in their associations with asthma and atopic diseases [21,22]. It seems some of these subtypes of sensitization may be ‘benign’ (i.e. not associated with clinical symptoms), and some are ‘pathologic’ [45], but we lack the tools at the point of care in primary practice, which would allow us to determine in individual sensitized patients whether sensitization is related to their atopic disease, or is just an incidental finding.

## COMPONENT-RESOLVED DIAGNOSTICS FOR ALLERGIC DISEASES

Traditionally, whole allergen extracts are used to diagnose allergic sensitization. However, we can now describe sensitization in much greater detail using component-resolved diagnostics (CRD, also known as molecular allergy tests) that measure sIgE response to a large number of allergenic molecules or allergen components (component-specific IgE, c-sIgE). For example, in allergy to peanut [46–48] and other foods [49,50], sensitization to some, but not all, allergenic proteins in allergen extracts is important for distinguishing true allergy from asymptomatic sensitization [51]. Consequently, CRD is firmly established in clinical practice in food allergy [52,53], but the data to support a similar approach in the field of asthma are lacking.

The field of molecular allergology is fast-moving, so the European Academy of Allergy and Clinical Immunology (EAACI) established a Task Force to summarize state-of-the-art information on allergen molecules, their clinical relevance and their application in diagnostic algorithms for clinical practice. The Task Force recently published The Molecular Allergology User's Guide 2.0, which provides comprehensive guidance on CRD for clinicians, scientists and interested readers [54^▪▪^]. Technological developments in molecular diagnostics have led to products in which sIgE responses to hundreds of allergen components from large number of different sources can be measured simultaneously using multiplex-based specific IgE antibody assays [55,56] (reviewed in [57^▪^,58^▪^]).

This increasing body of knowledge and technological capability has created conditions in which we can test the notion that assessing sensitization with CRD multiplex array data is more informative than standard allergy tests in respiratory allergy, and in particular that doing so would allow us to accurately identify clinically relevant sensitization. This has been indirectly confirmed in studies which have shown that several c-sIgEs to specific components in early life may be risk molecules for predicting asthma in school-age and adolescence [59,60^▪^], and that c-sIgE polysensitization to house dust mite (HDM) allergenic proteins predicts allergic disease [61].

## COMPONENT-RESOLVED DIAGNOSTICS AND ASTHMA

CRD arrays produce complex data sets, which can be interrogated by machine learning techniques [62^▪▪^,^63^]. To shed light on the relationship between allergic sensitization and respiratory diseases, we applied modern statistical and machine learning techniques to CRD microarray data [64–67]. Our initial analysis identified three patterns of c-sIgE response to 112 allergenic molecules measured by a commercial CRD array in mid-childhood, with a strong association between asthma and sensitization to a group of 27 components of plant, animal and fungal origin [65]. In further studies, we investigated temporal changes of c-sIgE responses to HDM and grass allergenic molecules from ages 5 to 11 years and demonstrated a clear association between different longitudinal trajectories of c-sIgE responses with clinical outcomes [66]. For instance, in relation to the risk of asthma diagnosis at age 11 years, the temporal difference was dominant in grass trajectories (early vs. late onset), with early-onset trajectory being associated with asthma diagnosis, and late-onset trajectory with rhinitis [66]. In a follow-up study that looked at the panel of 112 c-sIgEs longitudinally from infancy to adolescence, a grass/cat cluster (comprising c-sIgE to grass allergen Phl p 1 and cat allergen Fel d 1) at age 5 years was a strong predictor of asthma diagnosis at age 16 years [67].

However, whilst it is possible to ascertain the latent structure within the CRD multiplex array data using machine learning techniques, there are challenges both in developing this methodology into clinically useful tools for the diagnosis and prediction of asthma, and in giving biological interpretations to its results. A recent study has expanded these analyses to show that amongst sensitized individuals, a more detailed description of c-sIgE responses within each of the described component clusters – both in terms of the number of within-cluster c-sIgE responses and distinct c-sIgE patterns – adds potentially important information relevant to the clinical expression of symptoms [68^▪^]. This observation appeared to be of importance for both diagnostic and prognostic purposes, in that including the cumulative number of c-sIgEs within each component cluster improved the diagnosis and prediction of asthma and rhinitis, and that distinct within-cluster sensitization patterns differed in their association with health, asthma and rhinitis [68^▪^]. This should bolster our confidence that measuring sensitization using CRD arrays may be more informative than standard tests in respiratory allergy.

**FIGURE 1:**
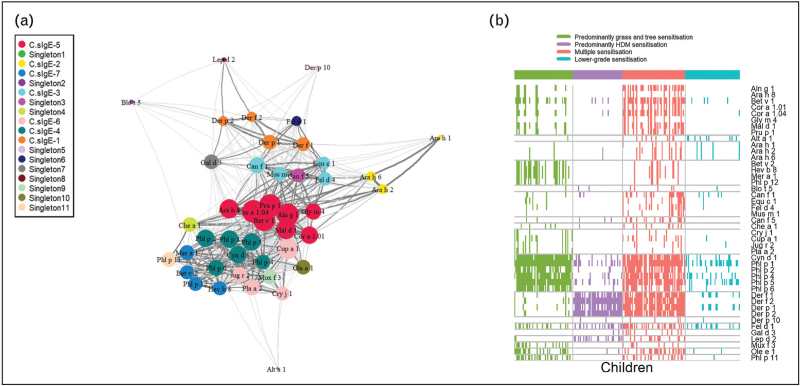
Specific IgE network and sensitization patterns in an unselected birth cohort. (a) Component-specific IgE network and hierarchical cluster reveal connectivity structure in sIgE. The network consists of a set of nodes, joined in pairs by lines or edges. Colours represent cluster memberships. (b) Patterns of IgE responses to allergen components for individual participants. Rows represent sIgEs, while columns indicate children. Colours represent sensitization clusters’ membership. Squares are coloured if and only if a child has a positive response, <0.30 to a particular sIgE. Adapted from [69].

Taking into account the findings of previous studies which showed that reducing the dimensionality of the multiplex array data by clustering components or patients produced stable, reproducible and meaningful clusters which differed in their association with the disease [64–67], but that additional valuable information could be gained by investigating within-cluster c-sIgE responses in terms of cumulative number of c-sIgE responses and distinct sensitization patterns [68^▪^], we developed a different framework to analysis of the complex CRD data. This involved applying network analysis to investigate interactions and connectivity patterns between c-sIgE on a CRD array, and in the first instance relating these to the presence of asthma (i.e. asthma diagnosis) [69]. It is important to emphasize that by clustering the c-sIgEs only, we identified seven clusters of responses, with cluster membership mapping closely to the structural homology of proteins and their biological source (Fig. 1a) [69], reflecting the results of other analyses using unsupervised clustering techniques [65–67].

When we clustered study participants, we identified four sensitization profiles that were characterized by unique patterns of sensitization to allergenic molecules from different component clusters [69]. These participant clusters were qualitatively labelled as multiple sensitization, with positive c-sIgE to multiple components across all seven component clusters; predominantly HDM sensitization; predominantly grass and tree sensitization; and lower grade sensitisation (Fig. 1b) [69]. Importantly, cluster membership was differentially associated with asthma risk. However, although a significantly higher proportion of children with asthma was found in the multiple sensitization and HDM clusters, the majority of children in each of these clusters did not have asthma [69]. To try to uncover the specific drivers of asthma risk, further machine learning based analysis was conducted, which showed that, in contrast to peanut allergy – in which sensitization to a specific peanut protein predicts clinical reactivity [46] – asthma is predicted not by c-sIgE to any individual molecule, but by the pattern of interaction between c-sIgEs [69]. Further analyses revealed a differential network of pairwise interactions between a limited number of c-sIgEs from different component clusters, which predicted asthma diagnosis with an excellent balance between sensitivity and specificity [69].

**FIGURE 2:**
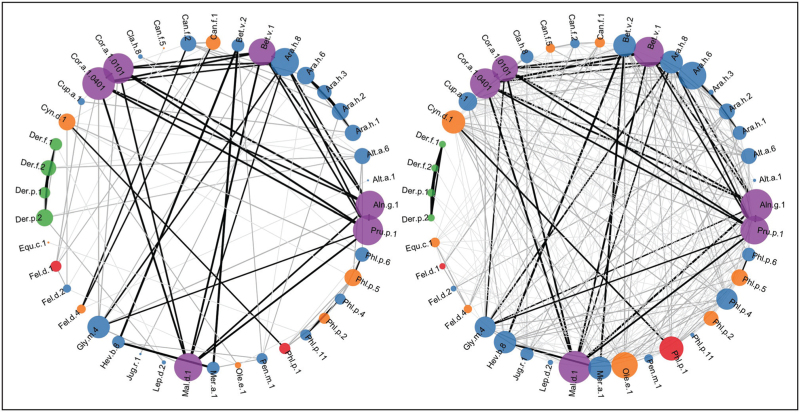
Correlation network for allergen component sensitization in mild/moderate versus severe asthma in the school-age cohort. Connections are based on Spearman rank-correlation coefficient (*r*). Pairs of sIgE components are connected if their correlation is significant (*P* < .05). Node colours represent allergen component cluster memberships, node diameter is proportional to the scaled connectivity of the specific component, while edge width represents the strength of correlation between pairs of sIgE components, while colours capture the differential connectivity. Different shades of gray indicate strength of associations: black edges (line) indicate *r* > 0.8, dark gray 0.6 < *r* < 0.8, gray 0.4 < *r* < 0.6 and light gray *r* < 0.4. Adapted from [70].

We then applied a similar approach to investigate whether, among sensitized patients with asthma, it would be possible to differentiate those with severe disease from patients with mild/moderate asthma [70]. These studies, carried out in the U-BIOPRED severe asthma cohort, demonstrated that the pattern of connectivity and interactions between c-sIgE to multiple allergenic proteins is a potentially important biomarker of asthma severity in sensitized school-age children and adults with asthma [70]. We showed that there is connectivity between a higher number of c-sIgEs in severe asthma, although these connections were weaker than those in mild asthma (Fig. 2); the mild asthma c-sIgE network had fewer co-sensitizations, but these were stronger [70].

Despite the fact that patients were recruited from seven different European countries with considerable differences in the pattern of allergen exposure and c-sIgE sensitization, we found remarkable consistencies in the connectivity structure among c-sIgEs in CRD arrays, and retrieved the same four sensitization profiles as is our previous studies at the UK general population level [70]. It is important to emphasize that all the above studies using different unsupervised learning approaches demonstrated a remarkable similarity in the structure of the CRD component sensitization patterns in the general population [65,67,69] and among patients with asthma [70], identifying clusters which were biologically plausible and reflected either the sources of allergenic proteins (e.g. grass and HDM clusters) [66] or the structural homogeneity of components within protein families (e.g. pathogenesis-related [PR]-10 and profilin clusters [67]). This provides important ‘sanity check’ to support the notion that it may be possible to develop algorithms to differentiate clinically important sensitization patterns associated with asthma diagnosis, prognosis and severity.

## CONCLUSION

Overall, the current evidence on the role of allergic sensitization in asthma suggests that:

(1) Sensitization is best thought of as an agglomerate of ‘sensitisation subtypes’.
(2) Some subtypes of allergic sensitization are ‘benign’ (i.e. not associated with clinical symptoms), and some are ‘pathologic’.
(3) Different sensitization subtypes may be associated with:
  (a) Amongst sensitized individuals of all ages, different clinical presentations (from no disease, asthma as a single disease, to allergic multimorbidity).
  (b) Amongst sensitized patients with asthma, with differing levels of asthma severity.
  (c) Amongst sensitized preschool children with and without lower airway symptoms, with different risk of subsequent asthma development.
(4) Interaction patterns between IgE antibodies to individual allergenic molecules (components) on CRD multiplex arrays may be hallmarks of the different sensitization subtypes relevant to asthma diagnosis, prognosis and severity.

This provides solid evidence that the use of machine learning based methodologies on complex CRD data generated from multiplex assays can help us to design better diagnostic tools to help practising physicians differentiate between benign and clinically important sensitization. However, major challenges related to achieving regulatory clearance of such multiplex chip-based assay [57^▪^,58^▪^], and the lack of interpretation algorithms for relevant clinical questions, need to be overcome to fully capitalize on these technologies.

## A WAY FORWARD

We propose that the identification of specific c-sIgE interaction patterns linked to asthma diagnosis, development and severity may be achieved through a combination of network analysis, and unsupervised and supervised statistical learning techniques.

Network analytics in general offers a powerful set of tools with which to analyse and visualize complex biological systems. Moreover, it assumes that biological processes are not controlled by individual components and disconnected linear pathways, but rather by a complex network of interactions. Understanding how these interactions give rise to biological processes and how these are pathogenically dysregulated will be crucial to understanding complex phenotypes in health and disease.

More specifically, differential network analysis may allow us to identify differences in the connectivity structure of c-sIgE responses from CRD data, which might act as hallmarks of asthma diagnosis and asthma severity. For prediction, the temporal evolution of interaction patterns and their role in asthma development might be investigated through dynamic network analysis to investigate time-varying connectivity structures and identify how the changes are related to disease development in individuals. This approach accounts for temporal evolution explicitly: it allows the evaluation of the dynamic patterns of connection in time with the changing status of individuals.

Supervised machine learning techniques, ranging from classical regression models to tree-based models and artificial neural networks, might allow us to identify CRD-based biomarkers. These techniques are set the goal of predicting a known output or target (such as asthma diagnosis, future risk or severity) and are given data which associate inputs (in this case, CRD data and its derivatives from network analysis) to these targets. From these particulars, the algorithms then ‘learn’ how the inputs – the interactions among c-sIgEs – relate to the outputs in general, potentially allowing us to identify which factors are important for differentiating different types of sensitization (benign and pathologic) and their relationship to asthma. However, to achieve this will require large international collaborations to guarantee validation and generalizability of any findings.

## Acknowledgements

*None.*

### Financial support and sponsorship

*D.C. is supported by the NIHR Imperial Biomedical Research Centre (BRC). The views expressed are those of the author(s) and not necessarily those of the NIHR or the Department of Health and Social Care.*

### Conflicts of interest

*Professor A. Custovic reports personal fees from Novartis, personal fees from Sanofi, personal fees from Stallergenes Greer, personal fees from AstraZeneca, personal fees from GSK, personal fees from La Roche-Posay, outside the submitted work. Other authors have nothing to disclose.*

## REFERENCES AND RECOMMENDED READING

Papers of particular interest, published within the annual period of review, have been highlighted as:

▪ of special interest
▪▪ of outstanding interest
